# Supplementary material for: Relationship between body mass index and clinical events in patients with atrial fibrillation undergoing percutaneous coronary intervention
Source: PLoS One. 2024 Sep 19;19(9):e0309758. doi: 10.1371/journal.pone.0309758 (PMC11412652; doi:10.1371/journal.pone.0309758)
Supplement: S1 Table — (DOCX) [file pone.0309758.s001.docx]

**Table S1. Multivariable Cox regression model of predictors for MACE**

| Variables | Hazard ratio | 95% CI | p value |
| --- | --- | --- | --- |
| Age (per year) | 1.03 | 0.99-1.05 | 0.08 |
| Male sex | 2.12 | 1.10-4.07 | 0.02 |
| Low BMI (< 21.3kg/m^2^) | 1.89 | 1.16-3.07 | 0.01 |
| Diabetes | 1.15 | 0.71-1.86 | 0.57 |
| Current smoking | 1.62 | 0.89-2.95 | 0.11 |
| Prior CABG | 2.04 | 0.98-4.24 | 0.06 |
| Peripheral artery disease | 1.82 | 0.96-3.47 | 0.07 |
| Prior heart failure | 1.03 | 0.60-1.76 | 0.92 |
| Severe CKD | 2.52 | 1.40-4.52 | 0.002 |
| Moderate to severe Anemia | 2.23 | 1.40-4.52 | 0.004 |
| Acute coronary syndrome | 1.46 | 0.90-2.35 | 0.12 |

BMI, body mass index; CABG, coronary artery bypass grafting; CKD, chronic kidney disease; CI, confidence interval; MACE, major adverse cardiovascular events.
