# Supplementary material for: Relationship between body mass index and clinical events in patients with atrial fibrillation undergoing percutaneous coronary intervention
Source: PLoS One. 2024 Sep 19;19(9):e0309758. doi: 10.1371/journal.pone.0309758 (PMC11412652; doi:10.1371/journal.pone.0309758)
Supplement: S2 Table — (DOCX) [file pone.0309758.s002.docx]

**Table S2. Multivariable Cox regression model of predictors for major bleeding events**

| Variables | Hazard ratio | 95% CI | p value |
| --- | --- | --- | --- |
| Male sex | 0.90 | 0.39-2.08 | 0.81 |
| Age (per year) | 1.04 | 0.99-1.09 | 0.12 |
| Low BMI (< 21.3kg/m^2^) | 0.99 | 0.45-2.18 | 0.97 |
| Severe CKD | 1.49 | 0.51-4.32 | 0.47 |
| Thrombocytopenia | 5.70 | 1.53-21.20 | 0.03 |
| Moderate to severe anemia | 2.56 | 1.15-5.75 | 0.03 |
| Using VKA at discharge | 0.59 | 0.19-1.83 | 0.34 |

BMI, body mass index; CKD, chronic kidney disease; CI, confidence interval; VKA, vitamin K antagonist.
