# Supplementary material for: Relationship between body mass index and clinical events in patients with atrial fibrillation undergoing percutaneous coronary intervention
Source: PLoS One. 2024 Sep 19;19(9):e0309758. doi: 10.1371/journal.pone.0309758 (PMC11412652; doi:10.1371/journal.pone.0309758)
Supplement: S3 Table — (DOCX) [file pone.0309758.s003.docx]

**Table S3. Adverse clinical events at 1 year in only men or women**

| Variables | Only men | | |  | Only women | | |
| --- | --- | --- | --- | --- | --- | --- | --- |
|  | Group 1  (n=132) | Group 2  (n=448) | p value |  | Group 1  (n=48) | Group 2  (n=92) | p value |
| NACE | 29 (22.0%) | 53 (11.8%) | 0.006 |  | 9 (18.8%) | 11 (12.0%) | 0.31 |
| MACE | 27 (20.5%) | 39 (8.7%) | <0.001 |  | 4 (8.3%) | 9 (9.8%) | 1.00 |
| All-cause death | 21 (15.9%) | 20 (4.5%) | <0.001 |  | 3 (6.3%) | 8 (8.7%) | 0.75 |
| Cardiovascular death | 11 (8.3%) | 9 (2.0%) | 0.002 |  | 1 (2.1%) | 6 (6.5%) | 0.42 |
| Myocardial infarction | 3 (2.3%) | 5 (1.1%) | 0.39 |  | 0 (0.0%) | 0 (0.0%) |  |
| Stent thrombosis | 1 (0.8%) | 4 (0.9%) | 1.00 |  | 0 (0.0%) | 0 (0.0%) |  |
| Ischemic stroke | 3 (2.3%) | 17 (3.8%) | 0.59 |  | 1 (2.1%) | 1 (1.1%) | 1.00 |
| Major bleeding (BARC 3 or 5) | 5 (3.8%) | 19 (4.2%) | 1.00 |  | 5 (10.4%) | 4 (4.4%) | 0.27 |
| All bleeding | 18 (13.6%) | 35 (7.8%) | 0.06 |  | 8 (16.7%) | 10 (10.9%) | 0.43 |

Values are expressed as n (%). BARC, Bleeding Academic Research Consortium; MACE, major adverse cardiovascular events; NACE, net adverse clinical events.
