# Supplementary material for: Relationship between body mass index and clinical events in patients with atrial fibrillation undergoing percutaneous coronary intervention
Source: PLoS One. 2024 Sep 19;19(9):e0309758. doi: 10.1371/journal.pone.0309758 (PMC11412652; doi:10.1371/journal.pone.0309758)
Supplement: S4 Table — (DOCX) [file pone.0309758.s004.docx]

**Table S4. Adverse clinical events at 1 year in the patients with <65 years or ≥65 years**

| Variables | Age <65 years | | |  | Age ≥65 years | | |
| --- | --- | --- | --- | --- | --- | --- | --- |
|  | Group 1  (n=13) | Group 2  (n=77) | p value |  | Group 1  (n=167) | Group 2  (n=463) | p value |
| NACE | 3 (23.1%) | 8 (10.4%) | 0.19 |  | 35 (21.0%) | 56 (12.1%) | 0.007 |
| MACE | 2 (15.4%) | 6 (7.8%) | 0.33 |  | 29 (17.4%) | 42 (9.1%) | 0.006 |
| All-cause death | 2 (15.4%) | 1 (1.3%) | 0.054 |  | 22 (13.2%) | 27 (5.8%) | 0.004 |
| Cardiovascular death | 1 (7.8%) | 0 (0.0%) | 0.14 |  | 11 (6.6%) | 15 (3.2%) | 0.07 |
| Myocardial infarction | 0 (0.0%) | 0 (0.0%) |  |  | 3 (1.8%) | 5 (1.1%) | 0.44 |
| Stent thrombosis | 0 (0.0%) | 1 (1.3%) | 1.00 |  | 1 (0.6%) | 3 (0.7%) | 1.00 |
| Ischemic stroke | 1 (7.7%) | 5 (6.5%) | 1.00 |  | 3 (1.8%) | 13 (2.8%) | 0.58 |
| Major bleeding (BARC 3 or 5) | 1 (7.7%) | 4 (5.2%) | 0.55 |  | 25 (15.0%) | 41 (8.9%) | 0.04 |
| All bleeding | 1 (7.7%) | 2 (2.6%) | 0.38 |  | 9 (5.4%) | 21 (4.5%) | 0.67 |

Values are expressed as n (%). BARC, Bleeding Academic Research Consortium; MACE, major adverse cardiovascular events; NACE, net adverse clinical events.
