# Supplementary material for: Relationship between body mass index and clinical events in patients with atrial fibrillation undergoing percutaneous coronary intervention
Source: PLoS One. 2024 Sep 19;19(9):e0309758. doi: 10.1371/journal.pone.0309758 (PMC11412652; doi:10.1371/journal.pone.0309758)
Supplement: S5 Table — (DOCX) [file pone.0309758.s005.docx]

**Table S5. Baseline characteristics after adjusted by age**

| Variables | Group 1  (n=177) | Group 2  (n=177) | p value |
| --- | --- | --- | --- |
| Age (years) | 77.1±8.3 | 77.1±8.2 | 0.98 |
| Male | 130 (73.4%) | 136 (76.8%) | 0.54 |
| Body weight (kg) | 50.6±7.4 | 65.8±10.3 | <0.001 |
| BMI (kg/m^2^) | 19.4±1.5 | 25.4±3.2 | <0.001 |
| Hypertension | 142 (80.2%) | 158 (89.3%) | 0.03 |
| Diabetes | 74 (41.8%) | 99 (55.9%) | 0.01 |
| Dyslipidemia | 116 (65.5%) | 141 (79.7%) | 0.004 |
| Current smoking | 22 (12.4%) | 30 (17.1%) | 0.23 |
| Family history of CAD | 16 (9.8%) | 21 (13.0%) | 0.39 |
| Prior angina pectoris | 45 (25.4%) | 47 (26.6%) | 0.90 |
| Previous MI | 44 (24.9%) | 41 (23.2%) | 0.80 |
| Prior PCI | 51 (28.8%) | 52 (29.4%) | 1.00 |
| Prior CABG | 14 (8.0%) | 9 (5.1%) | 0.29 |
| Hemodialysis | 7 (4.0%) | 2 (1.1%) | 0.17 |
| Paroxysmal AF | 92 (52.0%) | 99 (56.2%) | 0.46 |
| Peripheral artery disease | 13 (7.3%) | 18 (10.2%) | 0.45 |
| Prior heart failure | 59 (33.3%) | 46 (26.0%) | 0.16 |
| Prior major bleeding events | 15 (8.5%) | 13 (7.3%) | 0.84 |
| Severe CKD | 17 (9.6%) | 17 (9.6%) | 1.00 |
| Thrombocytopenia | 5 (2.8%) | 2 (1.1%) | 0.45 |
| Moderate to severe anemia | 44 (24.9%) | 21 (11.9%) | 0.002 |
| Liver cirrhosis | 3 (1.8%) | 0 (0.0%) | 0.12 |
| Active cancer | 15 (8.5%) | 10 (5.6%) | 0.41 |
| Prior bleeding stroke | 5 (2.8%) | 6 (3.4%) | 0.77 |
| Prior ischemic stroke | 36 (20.3%) | 44 (24.9%) | 0.37 |
| LVEF (%) | 49.5±15.2 | 52.2±14.4 | 0.11 |
| CHADS2 score | 2.8±1.3 | 3.0±1.3 | 0.14 |
| CHA2DS2-VASc score | 4.0±1.5 | 4.2±1.5 | 0.21 |
| HAS-BLED score | 3.3±1.0 | 3.3±0.9 | 0.66 |
| Laboratory data |  |  |  |
| Hemoglobin (g/dl) | 12.3±2.2 | 13.2±2.0 | <0.001 |
| Platelet (×10^4^/μL) | 21.6±10.7 | 19.0±5.1 | 0.005 |
| eGFR (ml/min/1.73 m^2^) | 52.5±20.4 | 52.8±17.3 | 0.89 |
| Total cholesterol (mg/dL) | 167.1±39.1 | 163.6±35.1 | 0.42 |
| HDL cholesterol (mg/dL) | 52.3±15.5 | 48.4±12.7 | 0.01 |
| LDL cholesterol (mg/dl) | 94.0±32.4 | 95.0±30.0 | 0.78 |
| Triglycerides (mg/dL) | 95.3±51.3 | 122.2±78.5 | <0.001 |
| Glycated hemoglobin (%) | 6.3±1.2 | 6.4±0.9 | 0.62 |
| Lesion and procedure |  |  |  |
| Acute coronary syndrome | 83 (46.9%) | 67 (37.9%) | 0.11 |
| Multivessel disease | 73 (41.5%) | 80 (45.2%) | 0.52 |
| Bifurcation lesion | 37 (21.0%) | 34 (19.3%) | 0.79 |
| Femoral approach | 26 (14.9%) | 35 (19.9%) | 0.26 |
| Mechanical support | 12 (6.8%) | 6 (3.4%) | 0.16 |
| DES type |  |  |  |
| Everolimus | 108 (61.0%) | 111 (62.7%) | 0.06 |
| Zotarolimus | 28 (15.8%) | 18 (10.2%) |  |
| Sirolimus | 30 (16.9%) | 27 (15.3%) |  |
| Biolimus | 5 (2.8%) | 3 (1.7%) |  |
| Multiple | 6 (3.4%) | 18 (10.2%) |  |
| Number of stents | 1.5±0.8 | 1.5±0.8 | 0.43 |
| Mean stent diameter (mm) | 2.9±0.5 | 3.0±0.5 | 0.36 |
| Total stent length (mm) | 36.4±22.3 | 38.1±23.0 | 0.47 |

Values are expressed as mean ± standard deviation or n (%). AF, atrial fibrillation; BMI, Body mass index; CABG, coronary artery bypass grafting; CAD, coronary artery disease; CKD, chronic kidney disease; DES, drug-eluting stent; eGFR, estimated glomerular filtration rate; HDL, high-density lipoprotein; LDL, low-density lipoprotein; LVEF, left ventricular ejection fraction; MI, myocardial infarction; PCI, percutaneous coronary intervention.
