# Supplementary material for: Relationship between body mass index and clinical events in patients with atrial fibrillation undergoing percutaneous coronary intervention
Source: PLoS One. 2024 Sep 19;19(9):e0309758. doi: 10.1371/journal.pone.0309758 (PMC11412652; doi:10.1371/journal.pone.0309758)
Supplement: S6 Table — (DOCX) [file pone.0309758.s006.docx]

**Table S6. Medications after adjusted by age**

| Variables | Group 1  (n=177) | Group 2  (n=177) | p value |
| --- | --- | --- | --- |
| Medication at discharge |  |  |  |
| Aspirin | 138 (78.0%) | 146 (82.5%) | 0.35 |
| P2Y12 inhibitor | 168 (94.9%) | 166 (93.8%) | 0.82 |
| VKA | 35 (19.8%) | 18 (10.2%) | 0.02 |
| DOAC | 140 (79.1%) | 157 (88.7%) | 0.02 |
| ACE-i/ARB | 103 (58.2%) | 120 (67.8%) | 0.08 |
| β-blocker | 130 (73.4%) | 126 (71.2%) | 0.72 |
| Statins | 143 (80.8%) | 147 (83.1%) | 0.68 |
| Oral antidiabetic agents | 43 (24.3%) | 56 (31.6%) | 0.16 |
| Insulin | 16 (9.0%) | 22 (12.4%) | 0.39 |
| PPI | 156 (88.1%) | 160 (90.4%) | 0.61 |
| Steroid | 8 (4.5%) | 3 (1.7%) | 0.22 |
| Medication at 6 months |  |  |  |
| Aspirin | 72 (46.5%) | 74 (46.2%) | 1.00 |
| P2Y12 inhibitor | 91 (58.7%) | 108 (67.5%) | 0.13 |
| OAC | 149 (96.1%) | 157 (98.1%) | 0.33 |
| Duration of triple therapy | 70.3±99.1 | 75.7±97.8 | 0.61 |

Values are expressed as mean ± standard deviation or n (%). ACE-i, angiotensin-converting enzyme inhibitor; ARB, angiotensin II receptor blocker; DOAC, direct oral anticoagulant; OAC, oral anticoagulant; PPI, proton pump inhibitor; VKA, vitamin K antagonist.
