# Supplementary material for: Relationship between body mass index and clinical events in patients with atrial fibrillation undergoing percutaneous coronary intervention
Source: PLoS One. 2024 Sep 19;19(9):e0309758. doi: 10.1371/journal.pone.0309758 (PMC11412652; doi:10.1371/journal.pone.0309758)
Supplement: S7 Table — (DOCX) [file pone.0309758.s007.docx]

**Table S7. Adverse clinical events at 1 year after adjusted by age**

| Variables | Group 1  (n=177) | Group 2  (n=177) | p value |
| --- | --- | --- | --- |
| NACE | 38 (21.5%) | 22 (12.4%) | 0.03 |
| MACE | 31 (17.5%) | 17 (9.6%) | 0.04 |
| All-cause death | 24 (13.6%) | 15 (8.5%) | 0.13 |
| Cardiovascular death | 12 (6.8%) | 9 (5.1%) | 0.65 |
| Myocardial infarction | 3 (1.7%) | 2 (1.1%) | 0.53 |
| Stent thrombosis | 1 (0.6%) | 1 (0.6%) | 0.74 |
| Ischemic stroke | 4 (2.3%) | 5 (2.8%) | 0.33 |
| Major bleeding (BARC 3 or 5) | 10 (5.6%) | 9 (5.1%) | 1.00 |
| All bleeding | 26 (14.9%) | 17 (9.6%) | 0.14 |

Values are expressed as n (%). BARC, Bleeding Academic Research Consortium; MACE, major adverse cardiovascular events; NACE, net adverse clinical events.
