# Supplementary material for: Relationship between body mass index and clinical events in patients with atrial fibrillation undergoing percutaneous coronary intervention
Source: PLoS One. 2024 Sep 19;19(9):e0309758. doi: 10.1371/journal.pone.0309758 (PMC11412652; doi:10.1371/journal.pone.0309758)
Supplement: S8 Table — (DOCX) [file pone.0309758.s008.docx]

**Table S8. Adverse clinical events at 1 year after excluding the patients with ACS or active cancer**

| Variables | Group 1  (n=86) | Group 2  (n=318) | p value |
| --- | --- | --- | --- |
| NACE | 16 (18.6%) | 31 (9.8%) | 0.04 |
| MACE | 11 (12.8%) | 22 (6.9%) | 0.12 |
| All-cause death | 9 (10.5%) | 16 (5.0%) | 0.08 |
| Cardiovascular death | 4 (4.7%) | 6 (1.9%) | 0.23 |
| Myocardial infarction | 0 (0.0%) | 2 (0.6%) | 1.00 |
| Stent thrombosis | 0 (0.0%) | 1 (0.3%) | 1.00 |
| Ischemic stroke | 2 (2.3%) | 7 (2.2%) | 1.00 |
| Major bleeding (BARC 3 or 5) | 5 (5.8%) | 13 (4.1%) | 0.55 |
| All bleeding | 15 (17.4%) | 25 (7.9%) | 0.01 |

Values are expressed as n (%). BARC, Bleeding Academic Research Consortium; MACE, major adverse cardiovascular events; NACE, net adverse clinical events.
