# Supplementary material for: Relationship between body mass index and clinical events in patients with atrial fibrillation undergoing percutaneous coronary intervention
Source: PLoS One. 2024 Sep 19;19(9):e0309758. doi: 10.1371/journal.pone.0309758 (PMC11412652; doi:10.1371/journal.pone.0309758)
Supplement: S9 Table — (DOCX) [file pone.0309758.s009.docx]

**Table S9. Adverse clinical events at 1 year in the patients with and without low-dose prasugrel**

| Variables | Patients with low-dose prasugrel | | |  | Patients without low-dose prasugrel | | |
| --- | --- | --- | --- | --- | --- | --- | --- |
|  | Group 1  (n=81) | Group 2  (n=271) | p value |  | Group 1  (n=99) | Group 2  (n=269) | p value |
| NACE | 12 (14.8%) | 37 (13.7%) | 0.85 |  | 26 (26.3%) | 27 (10.0%) | <0.001 |
| MACE | 10 (12.4%) | 31 (11.4%) | 0.84 |  | 21 (21.2%) | 17 (6.3%) | <0.001 |
| All-cause death | 8 (9.9%) | 16 (5.9%) | 0.22 |  | 16 (16.2%) | 12 (4.5%) | <0.001 |
| Cardiovascular death | 4 (4.9%) | 7 (2.6%) | 0.28 |  | 8 (8.1%) | 8 (3.0%) | 0.04 |
| Myocardial infarction | 1 (1.2%) | 2 (0.7%) | 0.55 |  | 2 (2.0%) | 3 (1.1%) | 0.61 |
| Stent thrombosis | 1 (1.2%) | 2 (0.7%) | 0.54 |  | 0 (0.0%) | 2 (0.7%) | 1.00 |
| Ischemic stroke | 0 (0.0%) | 13 (4.8%) | 0.045 |  | 4 (4.0%) | 5 (1.9%) | 0.26 |
| Major bleeding (BARC 3 or 5) | 3 (3.7%) | 12 (4.4%) | 1.00 |  | 7 (7.1%) | 11 (4.1%) | 0.28 |
| All bleeding | 10 (12.4%) | 27 (10.0%) | 0.54 |  | 16 (16.2%) | 18 (6.7%) | 0.01 |

Values are expressed as n (%). BARC, Bleeding Academic Research Consortium; MACE, major adverse cardiovascular events; NACE, net adverse clinical events.
