# Supplementary material for: Relationship between body mass index and clinical events in patients with atrial fibrillation undergoing percutaneous coronary intervention
Source: PLoS One. 2024 Sep 19;19(9):e0309758. doi: 10.1371/journal.pone.0309758 (PMC11412652; doi:10.1371/journal.pone.0309758)
Supplement: S10 Table — (DOCX) [file pone.0309758.s010.docx]

**Table S10. Adverse clinical events at 1 year in the patients classified by WHO criteria or median value**

| Variables | Using WHO criteria | | |  | Using median value | | |
| --- | --- | --- | --- | --- | --- | --- | --- |
|  | BMI <18.5 kg/m^2^  (n=44) | BMI ≥18.5 kg/m^2^  (n=676) | p value |  | BMI <24.0 kg/m^2^  (n=360) | BMI ≥24.0 kg/m^2^  (n=360) | p value |
| NACE | 9 (20.5%) | 93 (13.8%) | 0.26 |  | 61 (16.9%) | 41 (11.4%) | 0.04 |
| MACE | 7 (15.9%) | 72 (10.7%) | 0.31 |  | 46 (12.8%) | 33 (9.2%) | 0.15 |
| All-cause death | 6 (13.6%) | 46 (6.8%) | 0.12 |  | 31 (8.6%) | 21 (5.8%) | 0.19 |
| Cardiovascular death | 1 (2.3%) | 26 (3.9%) | 1.00 |  | 17 (4.7%) | 10 (2.8%) | 0.24 |
| Myocardial infarction | 0 (0.0%) | 8 (1.2%) | 1.00 |  | 4 (1.1%) | 4 (1.1%) | 1.00 |
| Stent thrombosis | 0 (0.0%) | 5 (0.7%) | 1.00 |  | 2 (0.6%) | 3 (0.8%) | 1.00 |
| Ischemic stroke | 1 (2.3%) | 21 (3.1%) | 1.00 |  | 10 (2.8%) | 12 (3.3%) | 0.83 |
| Major bleeding (BARC 3 or 5) | 3 (6.8%) | 30 (4.4%) | 0.45 |  | 20 (5.6%) | 13 (3.6%) | 0.28 |
| All bleeding | 8 (18.2%) | 63 (9.3%) | 0.07 |  | 45 (12.5%) | 26 (7.2%) | 0.02 |

Values are expressed as n (%). BARC, Bleeding Academic Research Consortium; BMI, body mass index; MACE, major adverse cardiovascular events; NACE, net adverse clinical events, WHO, world health organization.
